# Mental health, quality of life and self-management behaviours: online evaluation of inflammatory arthritis patients over 1 year of COVID-19 lockdowns

**DOI:** 10.1093/rap/rkad103

**Published:** 2023-11-29

**Authors:** Melissa Sweeney, Lewis Carpenter, Savia de Souza, Hema Chaplin, Hsiu Tung, Emma Caton, James Galloway, Andrew Cope, Mark Yates, Elena Nikiphorou, Sam Norton

**Affiliations:** Health Psychology Section, Institute of Psychiatry, Psychology and Neuroscience, King’s College London, London, UK; Health Psychology Section, Institute of Psychiatry, Psychology and Neuroscience, King’s College London, London, UK; Centre for Rheumatic Diseases, King’s College London, London, UK; Health Psychology Section, Institute of Psychiatry, Psychology and Neuroscience, King’s College London, London, UK; Health Psychology Section, Institute of Psychiatry, Psychology and Neuroscience, King’s College London, London, UK; Health Psychology Section, Institute of Psychiatry, Psychology and Neuroscience, King’s College London, London, UK; Centre for Rheumatic Diseases, King’s College London, London, UK; Centre for Rheumatic Diseases, King’s College London, London, UK; Centre for Rheumatic Diseases, King’s College London, London, UK; Centre for Rheumatic Diseases, King’s College London, London, UK; Rheumatology Department, King’s College Hospital, London, UK; Health Psychology Section, Institute of Psychiatry, Psychology and Neuroscience, King’s College London, London, UK; Centre for Rheumatic Diseases, King’s College London, London, UK

**Keywords:** inflammatory arthritis, COVID-19, quality of life, mental health, depression, anxiety

## Abstract

**Objective:**

Patients with inflammatory arthritis were especially vulnerable to the psychosocial and health impacts of coronavirus disease 2019 (COVID-19) and the lockdowns. This study investigated the impact of these changes on mental health, physical health and quality of life for inflammatory arthritis patients over 1 year following the initial lockdown in the UK.

**Methods:**

Three hundred and thirty-eight participants with inflammatory arthritis completed an ambidirectional study consisting of online questionnaires at four time points for 1 year. The questionnaires assessed demographic information, inflammatory arthritis condition, mental health, physical symptoms, self-management behaviours, COVID-19 status and impacts. Means, linear regressions and structural equation modelling for mediations were conducted over 12 months.

**Results:**

Physical health concerns peaked during June 2020, then declined, but did not return to baseline. Depression was associated with worse quality of life at baseline, as shown by the beta coefficient, (β= 0.94, *P* < 0.01), September (β = 0.92, *P* < 0.01), November (β= 0.77, *P* < 0.01) and 1 year (β = 0.77, *P* < 0.01). Likewise, anxiety was associated with worse quality of life at baseline (β = 1.92, *P* < 0.01), September (β = 2.06, *P* < 0.01), November (β = 1.66, *P* = 0.03) and 1 year (β = 1.51, *P* = 0.02). The association between depression and quality of life was mediated by physical activity (β= 0.13, *P* < 0.01) at baseline. The association between anxiety and quality of life was also mediated by physical activity (β = 0.25, *P* = 0.04) at baseline.

**Conclusion:**

Physical health continued to be worse 1 year later compared with before the COVID-19 lockdowns in patients with inflammatory arthritis. Mental health showed long-term effects on quality of life, with an impact for ≥12 months. Lastly, physical activity mediated between mental health and quality of life in the short term.

Key messages

- One year after COVID-19, inflammatory arthritis patients still have worse physical health.
- Mental health affects quality of life for ≥1 year.
- Physical activity mediates between mental health and quality of life.

## Introduction

The coronavirus disease 2019 (COVID-19) lockdowns affected populations around the world from March 2020 onwards. It has been estimated that the prevalence of mental health disorders rose by 8–34.7% during the lockdowns and has not returned fully to pre-COVID levels [1, 2]. Clinically vulnerable individuals, such as those with inflammatory arthritis, with increased risk of poor outcome of COVID-19 infection, were especially affected and experienced a greater elevation in risk of mental health impacts [3, 4]. Along with other clinically vulnerable groups, patients with inflammatory arthritis (a collection of chronic inflammatory autoimmune conditions) were recommended to follow varying levels of shielding or social distancing for long periods during 2020 and 2021 [5]. The effects of self-isolation on the disease activity, mental health and quality of life of inflammatory arthritis patients was unknown at the time of the initial lockdowns. Patients with inflammatory arthritis were already at increased risk of mental health problems and lower quality of life before the pandemic; therefore, ongoing monitoring of these factors through this period of social isolation and stress was needed [6, 7].

As the pandemic progressed, studies of inflammatory arthritis patients reported worsened mental health during the lockdowns, although most studies focused on short-term outcomes and results varied by country [8–12]. The lockdown restrictions also fluctuated in severity over the pandemic, but there have not been studies following patients over the long term to evaluate how symptoms changed through the relaxing and tightening of restrictions during the different lockdown periods. Research on the mental health and quality of life in inflammatory arthritis patients during the UK lockdowns can provide insight into whether these factors might affect the lives of patients in the long term. This could inform clinicians about symptoms and experiences of inflammatory arthritis patients in the aftermath of the pandemic in order that they can adjust to their care needs.

A prior study using initial data from this cohort examined changes in clinical care, mental health and physical health outcomes in inflammatory arthritis patients in the UK during the first 9 months of lockdowns [13]. Findings from the initial study showed that changes in clinical care owing to COVID-19 disruptions were associated with increased emotional distress, but only in the short term. It also found that worse mental health predicted worse physical health outcomes, with depression significantly affecting physical health for ≥5 months. A number of studies reported worsening of mental health for inflammatory arthritis patients [13–15], but several also demonstrated the impact of unique challenges, such as infection stress, social isolation and barriers to physical activity [8, 9, 12, 16]. The present study expands on the monitoring of physical health symptoms over time and examines the long-term changes in mental health, quality of life and self-management behaviours through the first year of the pandemic.

The objectives of this study were as follows: (i) to examine changes in self-reported health outcomes (disease activity, pain, fatigue and emotional distress) over time starting before the first lockdown through to 1 year after the end of the first lockdown; (ii) to determine the effects of mental health on quality of life over 1 year from the end of the first lockdown; and (iii) to identify behavioural mediators of the relationship between mental health and quality of life over the 12 month period.

## Methods

### Design and recruitment

The IA-COVID study was an online ambidirectional longitudinal mixed-methods series of questionnaires completed approximately every 3 months for 1 year from the end of the first lockdown to assess mental health, physical health and quality of life of inflammatory arthritis patients during the COVID-19 lockdowns in the UK. The baseline questionnaire was distributed in early June 2020, during a period of easing of restrictions. Follow-up questionnaires were distributed in early September 2020, late November 2020, early March 2021 and early June 2021. The follow-ups corresponded approximately to periods of lighter restrictions in September 2020, tighter restrictions during a second lockdown in November 2020, an easing of restrictions in March 2021, then lighter restrictions again in June 2021. Owing to an issue with the linkage of identification numbers, as a result of the survey being transferred between accounts owing to a change in institutional Qualtrics licence, it was not possible to include data from the March 2021 follow-up survey in the analysis.

Participants were recruited through social media and relevant charities. Eligibility criteria were as follows: age ≥18 years, living in the UK and having a self-reported inflammatory arthritis condition. The conditions included were RA, PsA, SpA, CTD and JIA. The questionnaire included only adults, but the JIA participants were classified according to their original diagnosis. Although the criteria specified that respondents must be residents in the UK, three respondents were included from crown dependencies that form part of the British Isles but are not in the UK. Informed consent was obtained from all participants for this study and additional related studies. Ethical approval was obtained from King’s College London Research Ethics Committee (LRS-19/20-18186). Informed consent was obtained online before the start of the questionnaire. Subsamples of participants were also included in a qualitative study and an ecological momentary assessment study [17].

### Measures

Topics covered by the questionnaire were as follows: details of condition, clinical care, self-management, disease outcomes, mental health, quality of life, COVID-19 clinical information and COVID-19 experience. All questions were self-report.

#### Arthritis symptoms and quality of life

Visual analogue scales (VASs) were completed for the previous week for patient global assessment of disease activity (PGA), pain and fatigue at each time point. The baseline survey also included retrospective assessments of pre-lockdown and peri-lockdown time points: first week of March 2020 and first week of April 2020. Visual analogue scales are commonly used in rheumatic conditions and considered to be appropriate measures for intensity of an experience, such as disease activity and pain [18]. The VASs for PGA and fatigue, for example, both have an intraclass correlation coefficient (ICC) for reliability of 0.74 [19].

In addition to VAS measures, the musculoskeletal health questionnaire (MSK-HQ) was completed at each time point, but not retrospectively. The MSK-HQ is a 14-item tool that measures the impact of disease on various aspects of wellbeing, such as washing and dressing, sleep and emotional functioning. However, in this study it was shortened to 12 questions for brevity of the overall questionnaire by removing questions about emotional wellbeing and fatigue because they were covered by other questions. The MSK-HQ has been demonstrated to have high reliability (ICC = 0.84) and good validity in relationship to other measures [20].

#### Lifestyle measures

Inflammatory diet was evaluated by a shortened healthy eating assessment, which is an eight-item food frequency questionnaire that evaluates dietary patterns. It has been shown to have a moderate correlation compared with the National Cancer Institute (NCI) screener (*r* = 0.39) [21, 22]. The baseline questions also compared how the diets of participants changed from before the COVID-19 lockdowns. Physical activity was evaluated with one question (‘How many hours did you spend sitting or lying down during the daytime per day on average?’) modified from the international physical activity questionnaire (IPAQ), a valid (*r* = 0.9) and reliable (ICC = 0.67–0.81) measure [23].

#### Mental health measures

As with the disease outcome measures, emotional distress in the past week was measured with a VAS at each time point and retrospectively in the baseline questionnaire. Depressive symptoms were evaluated with the personal health questionnaire depression scale (PHQ-8). The PHQ-8 is a shortened version of the PHQ-9 scale, omitting the item regarding suicidal ideation, and has been validated for use as a depression screening tool in various contexts [24]. Anxiety symptoms were assessed by the GAD-2, which is the first two questions from the generalized anxiety disorder assessment (GAD-7) and has shown good sensitivity (89%) and specificity (82%) [25].

### Statistical analysis

The mean VAS scores for PGA, pain, fatigue and emotional distress were calculated for each time point, including retrospectively at pre- and peri-lockdown (March and April 2020, respectively). The means for the PHQ-8 and MSK-HQ were also calculated. Plots of these means over time were created. Mixed-model regressions were run to examine the effects of baseline PHQ and GAD scores on MSK-HQ at each follow-up, controlling for confounders of age, sex and inflammatory arthritis condition.

Structural equation models were then used to examine mediators between PHQ/GAD scores and MSK-HQ outcomes, using the PHQ or GAD score at the prior visit to predict the MSK-HQ at the subsequent visit. Behavioural factors of inflammatory diet and physical activity at the prior visit were used as mediators. The models controlled for age, sex and condition. These were conducted for the baseline and each follow-up. Student’s unpaired *t*-tests were conducted to determine differences in demographics between participants who were included in the sample compared with those who dropped out of the study. All analyses were conducted in STATA v.17.0 (StataCorp LLC, College Station, TX).

## Results

**Figure 1.**
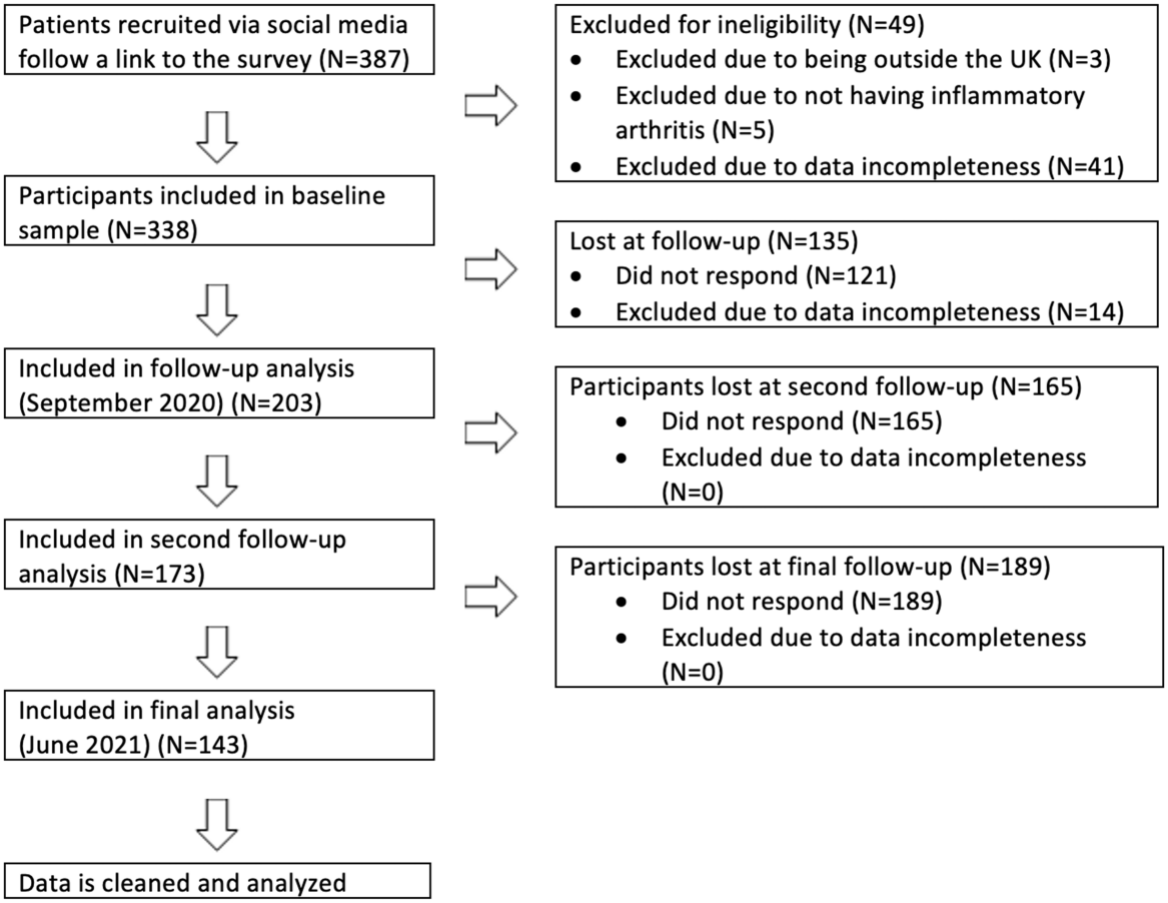
Recruitment flowchart

Table 1 displays the baseline characteristics of the sample by inflammatory arthritis condition. A total of 338 participants completed the baseline assessment in June 2020. Fig. 1 shows a flowchart of the recruitment process. Data were available for 203 (60.0%), 173 (51.2%) and 143 (42.3%) participants at the September 2020, November 2020 and June 2021 follow-ups, respectively. The analysis sample included 260 (77.0%) participants who completed the baseline survey and at least one follow-up survey. The sample was mostly female (90.2%), White (97.5%), and had an average age of 47.9 years old, with an age range of 19–77 years. Supplementary Table S1, available at *Rheumatology Advances in Practice* online, displays χ^2^ tests comparing demographics of participants included in the study with participants who dropped out, with the only difference being that younger participants were more likely to drop out. Thus, only age appeared to be affected by bias from the dropout at follow-ups.

**Table 1.** Demographics of the sample

Characteristic	Total sample	RA	PsA	SpA	CTD	JIA
*n*	338	100	98	50	85	5
Age, mean (s.d.), years	47.90 (13.64)	53.06 (13.37)	46.39 (11.97)	41.18 (12.09)	48.68 (12.67)	28.2 (11.52)
Female sex, %	90.2	92.0	85.7	82.0	97.6	100
Education, %						
No formal qualifications	3.5	2.0	3.1	4.0	5.9	0.0
O level or GCSE	21.3	22.0	23.5	16.0	21.2	20.0
A level	21.0	23.0	25.5	16.0	16.5	20.0
Undergraduate degree	32.2	27.0	30.6	45.0	31.8	40.0
Postgraduate degree	21.9	26.0	17.4	18.0	24.7	20.0
Baseline social distancing, %						
None of the time	1.58	3.1	2.1	0	0	0
Some of the time	4.75	2.1	8.6	6.6	1.2	25
Most of the time	39.56	41.5	43.0	37.8	33.7	50
All the time	54.11	53.2	46.2	55.6	65.0	25.0

### Physical and mental health

**Figure 2.**
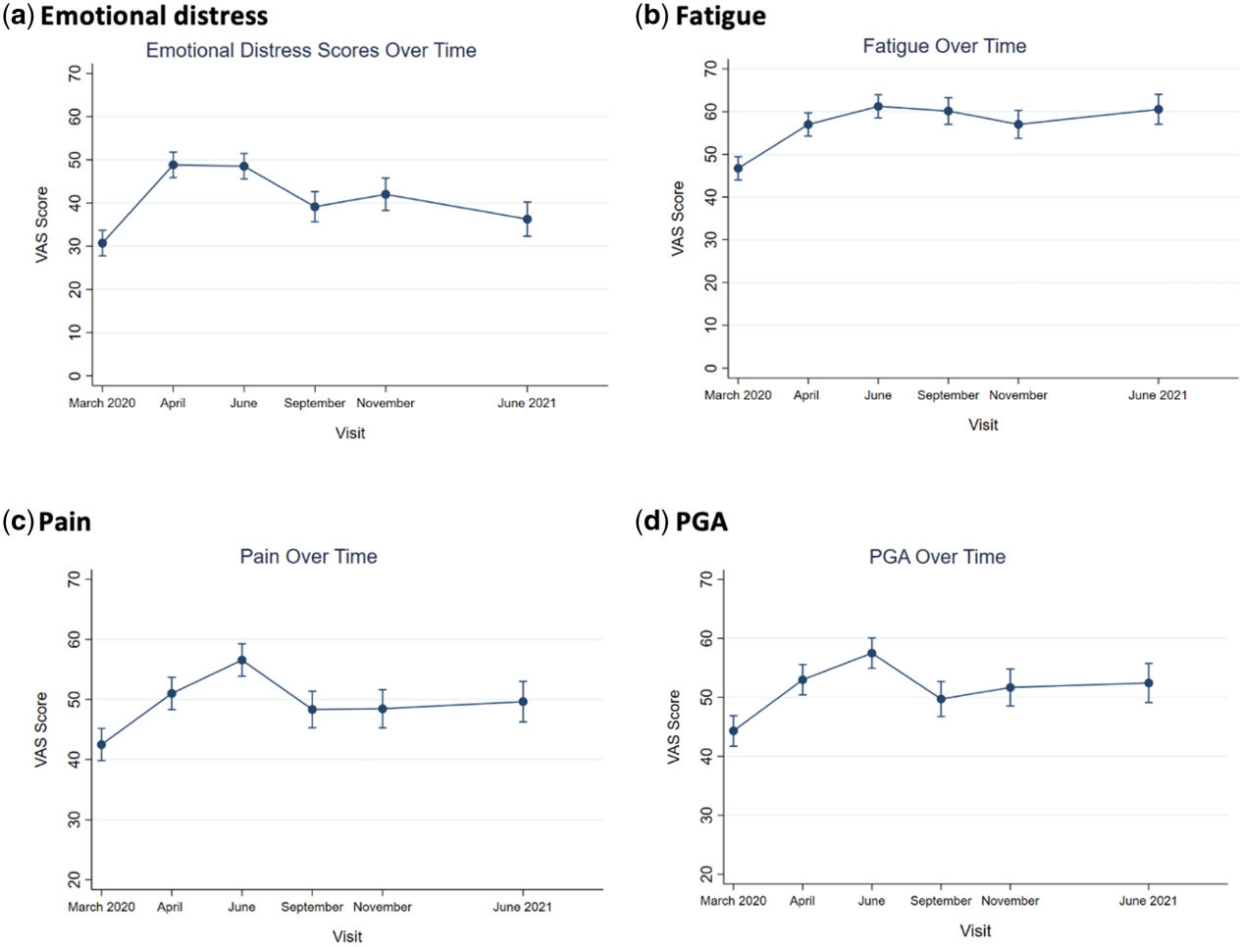
Visual analogue scale scores from pre-lockdown March 2020 to June 2021

The plots of the mean VAS scores from March 2020 to June 2021 are shown in Fig. 2. For Patient Global Assessment, pain, fatigue and emotional distress, scores were all increased during the initial lockdown (shown in the initial upward trend in panels a–d) compared with retrospectively reported pre-lockdown levels. Levels of PGA, pain and distress then improved slowly over the following 12 months, but without returning to pre-lockdown levels. This slight increase is also shown across all of the panels a–d. Fatigue levels remained high during the 12 months of follow-up.

**Figure 3.**
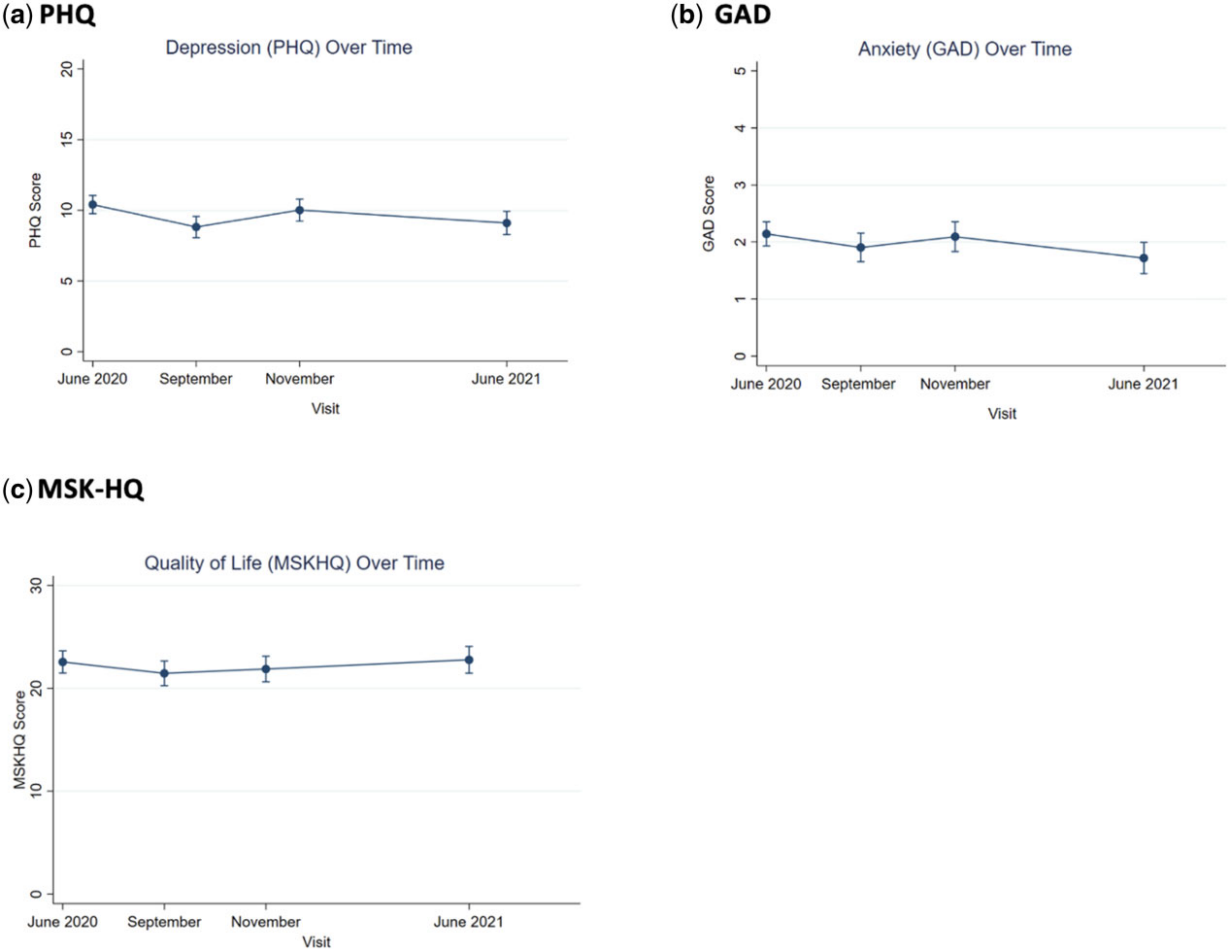
Mean mental health and quality of life scores from June 2020 to June 2021

Fig. 3A–C shows the mean scores for the depression (PHQ-8), anxiety (GAD-2) and quality of life (MSK-HQ) (Fig. 3A–C, respectively) over 12 months following the initial lockdown. Both depression and anxiety symptoms declined slightly over time, but were mostly stable, as shown in panels a and b which show slight downturns in scores. The MSK-HQ also stayed fairly stable throughout the year of lockdowns, as displayed in panel c showing little variation in scores over time.

### Quality of life

Linear mixed-effects models were used to assess the association between baseline mental health and MSK-HQ over time. Table 2 displays the coefficients of the mixed-effects models at each time point. All the time points were significant, but the larger beta coefficients indicate a stronger relationship with the MSK-HQ. Additionally, all the results have positive beta coefficients, indicating that as mental health scores increased, the MSK-HQ scores increased accordingly. Greater depressive symptoms (PHQ-8) at baseline were associated with worse quality of life (MSK-HQ) at all time points over 1 year, even after controlling for age, gender and condition. Likewise, higher anxiety symptoms (GAD-2) at baseline were also associated with worse quality of life at all time points throughout the year, again controlling for age, sex and condition.

**Table 2.** Regression coefficients for mental health factors on musculoskeletal health questionnaire scores over 1 year

	June 2020	*P*-value	September 2020	*P*-value	November 2020	*P*-value	June 2021	*P*-value
Baseline PHQ	0.94	<0.01	0.92	<0.01	0.77	<0.01	0.70	<0.01
Baseline GAD	1.92	<0.01	2.06	<0.01	1.66	0.03	1.51	<0.01

GAD: generalized anxiety disorder assessment; PHQ: personal health questionnaire depression scale.

### Behavioural mediators

**Figure 4.**
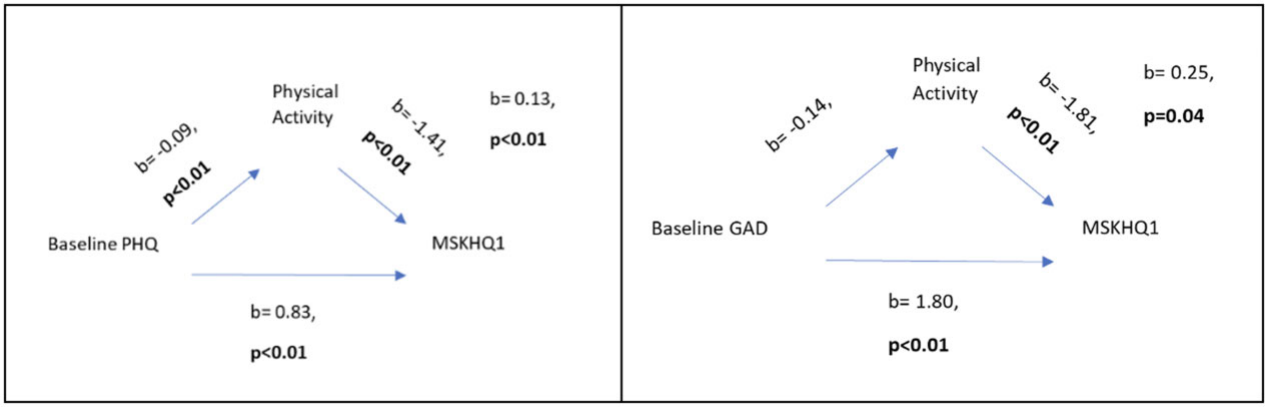
Mediations

The relationship between depression (PHQ) in June 2020 and quality of life (MSK-HQ) in September 2020 was mediated by physical activity (β = 0.13, *P* <* *0.01) but not diet at baseline (Fig. 4). The small beta coefficient indicates that although the mediating relationship of physical activity between depression and the MSK-HQ was significant, it was not a strong effect; therefore, physical activity was a mediator, but its role was not large. Likewise, the relationship between anxiety in June 2020 and quality of life (MSK-HQ) in September 2020 was also mediated by physical activity (β = 0.25, *P* = 0.04) but not diet at baseline. This beta coefficient was slightly larger, indicating a larger effect compared with the mediating role of physical activity between depression and the MSK-HQ.

The later follow-ups did not show any significant mediations by diet or physical activity between the depression score and quality of life at the following time point. Anxiety scores also showed no significant mediations after the baseline time point.

## Discussion

Mental health for inflammatory arthritis patients during the COVID-19 lockdowns appears to have a long-lasting and complex relationship with quality of life, physical symptoms and self-management behaviours. Our previous publication showed that mental health had worsened during the first several months of COVID-19 lockdowns [13], and the present results show that this effect persisted for ≥12 months, with impacts on quality of life.

Emotional distress nearly returned to baseline after 1 year, whereas physical symptoms remained elevated. This is reflective of our previous study, showing delayed effects on physical health continuing, although diminishing over time, in addition to other studies showing that physical health can remain impacted by mental health in the longer term [13, 26, 27]. Although the PHQ and GAD were not collected retrospectively at the baseline for March and April 2020, the emotional distress VAS score suggests that scores peaked in April–June 2020, then slowly decreased. This might be reflective of people adapting to the COVID-19 uncertainty and lockdown stressors as people developed new coping skills and routines despite the ongoing changes [28].

The relationship between mental health and quality of life over time was consistent for both depression and anxiety, in that worse depression and anxiety were both linked with later worse quality of life. Given that the MSK-HQ has several questions about routine, such as sleep, work and socializing, it is likely that those scores would be affected by depression and anxiety, given the overlap in symptoms or areas of life. However, the long-lasting impact of baseline depression or anxiety on quality of life a year later is notable and consistent with another study on mental health and quality of life during lockdowns in this population [29]. The impact does decrease over time, but the persistence of influence is important for clinicians to be aware of for patients recovering from lockdown stressors. There are few studies that have investigated the longitudinal impact of mental health on quality of life in this population, but the results are in line with prior studies also showing long-term effects of mental health on quality of life and self-management [30, 31].

Finally, physical activity was shown to mediate between depression and quality of life at baseline, but not at later time points. This might be because emotional distress, depression and anxiety all peaked at the start of the lockdowns, then gradually decreased as people seemed to adjust over time; therefore, the impacts of mediators might have been more amplified at that time and faded alongside emotional distress. Alternatively, as restrictions loosened, other possible mediators, such as increased social support, might have taken on a stronger influence. Finally, 70% of participants made changes in physical activity at baseline, and the effects of the changes would probably have been most apparent initially, then faded as people became accustomed to the changes. A fading of the effect of changes in self-management behaviours on quality of life would be important for clinicians to note in order that they can encourage patients to maintain healthy habits in the long term for the benefits on physical health outcomes. Future research could also include more detailed questionnaires about physical activity and changes to elucidate the nuances better.

In contrast, diet was not a significant mediator at any time point. This might be because of the complexity of measuring diet accurately compared with physical activity, such as measuring the frequency of items eaten, participants’ memory of them, and variety in food product quality. Furthermore, the measure used for diet was short compared with other measures, such as food frequency questionnaires, which would lead to more thorough assessment and thus more accurate results. Future research with a greater focus on diet would benefit from more extensive measurement. It is also possible that diet and physical activity have different mechanisms or time frames in which they exert an effect.

This study benefitted from having several follow-ups over a relatively long period of time. Future research could investigate further whether there are effects that manifest beyond 1 year. The study also had a large sample size. Although there was some bias in gender, age and ethnicity, it did include a range of inflammatory arthritis conditions. Although bias was tested for in dropout and was found only to affect age, the original sample could still contain bias from the baseline. The most notable bias was in sex. Although there is a higher rate of inflammatory arthritis conditions in women, the online format of the study appears to have resulted in a self-selection bias towards female participants. Thus, the results might not be generalizable to men, because they were a small minority of the sample. Additionally, the measures were all self-reported, and some were limited further by also being retrospective. The pre- and peri-lockdown questions were completed retrospectively and might have differences compared with those collected non-retrospectively. Recall bias in retrospective questions appears to be especially true for affective experiences; therefore, these results should be interpreted more cautiously in comparison to those obtained non-retrospectively. Owing to the recruitment being online during COVID-19, the diagnoses were self-reported; therefore, there is also a limitation in the inability to validate the disease activity or diagnoses. Lastly, many of the questions on the questionnaire were shortened or adapted to the context of COVID-19; therefore, they might not reflect the same validity as other contexts.

Overall, the present study has confirmed some of the findings from our earlier analysis, such as the persistence of worsened mental and physical health symptoms in inflammatory arthritis patients during the COVID-19 lockdowns. The additional analyses also highlighted the importance of distinguishing between mental health measures in terms of anxiety *vs* depression in future inflammatory arthritis studies, owing to possible differences in outcomes. Future research could be completed with objective clinical measures that could validate the results found in this study. Although inflammatory arthritis patients seem to be recovering over time from the stressors that affected their mental and physical health, clinicians should keep in mind that they might still be presenting with worsened symptoms and might need additional support in the longer term.

## Supplementary Material

rkad103_Supplementary_Data

## Data Availability

The data underlying this article will be shared on reasonable request to the corresponding author.
