# Supplementary material for: Mental health, quality of life and self-management behaviours: online evaluation of inflammatory arthritis patients over 1 year of COVID-19 lockdowns
Source: Rheumatol Adv Pract. 2023 Nov 29;8(1):rkad103. doi: 10.1093/rap/rkad103 (PMC10713280; doi:10.1093/rap/rkad103)
Supplement: rkad103_Supplementary_Data [file rkad103_supplementary_data.docx]

**Supplementary Table S1. Chi-squared tests for participants included vs participants who dropped out.**

|  | **Total Sample** | **Included in sample** | **Not included in sample (drop out)** | **p** |
| --- | --- | --- | --- | --- |
| **N** | 338 | 260 | 78 |  |
| **Age, Mean (SD)** | 47.90 (13.64) | 50.54 (14.05) | 45.96 (13.02) | **P=0.03** |
| **Gender, %** | 90.2% | 93.7% | 87.7% | P=0.07 |
| **Education, %** |  |  |  | P=0.48 |
| No formal qualifications | 3.5% | 3.5% | 3.6% |  |
| O-level, GCSE or equivalent | 21.3% | 19.6% | 22.6% |  |
| A-level or equivalent | 21.0% | 23.1% | 19.5% |  |
| Undergraduate degree or equivalent | 32.2% | 32.2% | 32.3% |  |
| Postgraduate degree or equivalent | 21.9% | 21.7% | 22.1% |  |
